# Genetic Variants and Functional Analyses of the *ATG16L1* Gene Promoter in Acute Myocardial Infarction

**DOI:** 10.3389/fgene.2021.591954

**Published:** 2021-06-17

**Authors:** Falan Han, Shuchao Pang, Zhaoqing Sun, Yinghua Cui, Bo Yan

**Affiliations:** ^1^Cheeloo College of Medicine, Shandong University, Jinan, China; ^2^Shandong Provincial Key Laboratory of Cardiac Disease Diagnosis and Treatment, Affiliated Hospital of Jining Medical University, Jining Medical University, Jining, China; ^3^Division of Cardiology, Affiliated Hospital of Jining Medical University, Jining Medical University, Jining, China; ^4^The Center for Molecular Genetics of Cardiovascular Diseases, Affiliated Hospital of Jining Medical University, Jining Medical University, Jining, China; ^5^Shandong Provincial Sino-US Cooperation Research Center for Translational Medicine, Affiliated Hospital of Jining Medical University, Jining Medical University, Jining, China

**Keywords:** acute myocardial infarction, *ATG16L1*, gene promoter, single-nucleotide polymorphisms, autophagy

## Abstract

**Background:**

Acute myocardial infarction (AMI), a common complex disease caused by an interaction between genetic and environmental factors, is a serious type of coronary artery disease and is also a leading cause of death worldwide. Autophagy-related 16-like 1 (*ATG16L1*) is a key regulatory factor of autophagy and plays an important role in induced autophagy. In the cardiovascular system, autophagy is essential to preserve the homeostasis and function of the heart and blood vessels. No studies have hitherto examined the association between AMI and *ATG16L1* gene promoter.

**Methods:**

We conducted a case-control study, using polymerase chain reaction and sequencing techniques, dual luciferase reporter assay, and electrophoretic mobility shift assay, to analyze genetic and functional variation in the *ATG16L1* gene promoter between AMI and controls. A variety of statistical analyses were used to analyze the allele and genotype frequencies and the relationship between single-nucleotide polymorphisms (SNPs) and AMI.

**Results:**

In all, 10 SNPs and two DNA-sequence variants (DSVs) were identified in 688 subjects, and three *ATG16L1* gene promoter mutations [g.233250693 T > C (rs185213911), g.233250946 G > A (rs568956599), g.233251133 C > G (rs1301744254)] that were identified in AMI patients significantly altered the transcriptional activity of *ATG16L1* gene promoter in HEH2, HEK-293, and H9c2 cells (*P* < 0.05). Further electrophoretic mobility shift assays indicated that the SNPs affected the binding of transcription factors (*P* < 0.01).

**Conclusion:**

*ATG16L1* gene promoter mutations in AMI patients may affect the binding of transcription factors and change the transcriptional activity of the *ATG16L1* gene, changing the level of autophagy and contributing to the occurrence and development of AMI as rare and low-frequency risk factors.

## Introduction

Acute myocardial infarction (AMI) is a serious type of coronary artery disease (CAD) and is a leading cause of death worldwide (Lopez et al., 2006). CAD is a common complex disease resulting from an interaction between genetic and environmental factors (Yusuf et al., 2004). Accepted and suspected risk factors include age, smoking, hyperuricemia, hypertension, hyperhomocysteinemia, obesity, diabetes, triglyceridemia, hypercholesterolemia, and psychosocial stress (Broeckel et al., 2002). Genome-wide association studies have identified about 152 loci associated with AMI and CAD in humans (Deloukas et al., 2013; Khera and Kathiresan, 2017). Most of these variants are in non-coding regions and are co-inherited with hundreds of candidate regulatory variants (Miller et al., 2016). CAD is a complex disease that is affected by DNA variants at numerous loci distributed throughout the genome (Musunuru and Kathiresan, 2019). These mutations usually have little effect and do not drive a classic Mendelian inheritance pattern within a family, so large-scale, population-based association studies are needed to examine them closely (Musunuru and Kathiresan, 2019). Atherosclerosis is an inflammatory disease that interacts with immune mechanisms and is also a main cause of CAD (Wolf and Ley, 2019). Inflammation plays a key role in CAD, acute coronary events, and other manifestations (Hansson, 2005; Watkins and Farrall, 2006; Newby, 2019). Autophagy proteins contribute to the functioning of virtually all cell types involved in inflammation (Matsuzawa-Ishimoto et al., 2018). It plays an important role in both innate and adaptive immunity. Some studies have shown that autophagy genes are associated with inflammatory diseases (Kuballa et al., 2012). Autophagy also plays an important role in inflammation and the inhibition of apoptosis, and has a protective effect in atherosclerosis, acting as a cholesterol efflux promoter (Martinet and De Meyer, 2009; Ouimet et al., 2011; Razani et al., 2012). Autophagy has also been demonstrated to have a protective effect in the cardiac ischemic response. In addition, the inhibition of autophagy leads to adverse cardiac remodeling after AMI (Wang et al., 2018). *ATG16L1* is a well-known key regulator of autophagy. To identify new loci that influence the progress of AMI, we compared genetic and functional variation in the *ATG16L1* gene promoter between AMI patients and healthy people.

Macroautophagy is often simply referred to as autophagy, a conservative process transported to the lysosome through a double-membrane-bound vesicle intermediate; it is a central catabolic process that is essential for cell homeostasis under basal and stress conditions and has broad implications (Dikic and Elazar, 2018; Leidal et al., 2018; Schütter et al., 2020). Autophagy is catabolic for the lysosomal degradation of cytosolic components in eukaryotes and has been implicated in physiological and pathological processes in several human diseases (Mizushima et al., 2010). An increasing body of evidence indicates that dysfunction in autophagy is part of various metabolic disorders, including obesity, diabetes, atherosclerosis, and non-alcoholic fatty liver disease (Ueno and Komatsu, 2017; Zhang et al., 2018). It is mediated by evolutionarily conserved autophagy-related genes, and it selectively targets dysfunctional organelles, intracellular microorganisms, and pathogenic proteins that may lead to disease. There is a clear etiological link between the mutations that control autophagy and human diseases, particularly neurodegenerative diseases, inflammatory diseases, and cancer (Levine and Kroemer, 2019). Autophagy is mediated by a large group of autophagy-related proteins (ATGs). Their roles include autophagy induction, autophagosome formation and extension, autophagolysosome formation, and the degradation of contents (Hansen et al., 2018). More than 30 kinds of ATGs have been found to regulate autophagy. *ATG16L1* contains an N-terminal ATG5-binding domain and a middle coiled-coil domain (CCD) that mediates homodimerization (Xu et al., 2019b). It forms the ATG12–ATG5–*ATG16L1* dimer with ATG12 and ATG5, which are essential for proper extension of the isolation membrane (Levine et al., 2011; Scrivo et al., 2019). *ATG16L1* has the inherent ability to bind to lipids, which plays an important role in the process of LC3 liposomeization and autophagosome maturation (Dudley et al., 2019). In addition, *ATG16L1* precursor homofusion is a key event in the early stage of cell autophagy, which combines the acquisition of cell membranes with the formation of autophagosomes and regulates the size of vesicles (Moreau et al., 2011).

Studies have shown that coding variation in *ATG16L1* is related to autophagy and endoplasmic reticulum (ER) dysfunction. *ATG16L1* deficiency aggravates the harmful effects of IL-22 signal transduction and leads to excessive death of epithelial cells (Aden et al., 2018). This cytoprotective function of *ATG16L1* is associated with the role of autophagy in promoting mitochondrial homeostasis (Matsuzawa-Ishimoto et al., 2017). *ATG16L1* is necessary for lysosomal exocytosis and the formation of plasma-membrane vesicles, and it promotes toxin resistance and inhibits Lm cell proliferation by promoting plasma-membrane repair. *ATG16L1* deficiency can lead to an accumulation of cholesterol in cells, damage to cell-membrane repair, and the exocytosis of lysosomes, which can lead to defects in membrane repair (Tan et al., 2018). *ATG16L1* is a target of the PKA activity of endothelial cells. The phosphorylation of *ATG16L1* reduces the autophagy of endothelial cells. PKA activity promotes angiogenesis by restricting the phosphorylation of *ATG16L1* (Zhao et al., 2019). The reversible phosphorylation of *ATG16L1* plays an important role in the regulation of hypoxia/reoxygenation (H/R) in autophagy. H/R can increase autophagy and promote the phosphorylation of *ATG16L1* in cultured cardiomyocytes, which may have a role in protecting cardiomyocytes from apoptosis under metabolic stress (Song et al., 2015). Although there have been some studies and reports on *ATG16L1* in the context of immunity, atherosclerosis, and cardiomyocytes, there has been no work on the role of the *ATG16L1* gene in the occurrence and development of CAD or AMI. DNA-sequence variants (DSVs) and single-nucleotide polymorphisms (SNPs) in the promoter of the *ATG16L1* gene in patients with AMI have not been studied or reported. We speculate that mutations in the *ATG16L1* gene promoter may lead to abnormal gene expression, and this may play an important role in the occurrence and development of coronary heart disease and AMI. Therefore, we investigated gene mutations in the promoter region of *ATG16L1* in AMI and studied its effects on the transcription activity of the *ATG16L1* gene and its potential mechanism for the formation and progress of AMI.

In this innovative study, genetic and functional variation in the *ATG16L1* gene promoter was evaluated in AMI patients and in normal populations across China for the first time.

## Materials and Methods

### Study Subjects

A randomly selected experimental group of 329 patients (237 males and 92 females; mean age: 63.80 ± 12.27 years) with clinically confirmed AMI was recruited from the Affiliated Hospital of Jining Medical College from November 2012 to February 2017. At the same time, we recruited a control group of 359 healthy controls (229 males and 130 females; mean age: 45.76 ± 12.77 years) with no family history of coronary heart disease, of the same race, and with no blood relationship with AMI patients. The AMI inclusion criteria were as follows: typical clinical manifestations of AMI; changes in electrocardiogram and dynamic evolution; changes in the serum myocardial necrosis markers cTnT, cTnI, and CK-MB; and results for echocardiography, radionuclide myocardial perfusion imaging, coronary angiography, and other auxiliary examinations meeting the international standard for the diagnosis of AMI. Exclusion criteria were as follows: history of heart valve disease, aortic dissection, cardiomyopathy, myocarditis or angioplasty, or tumor. All subjects provided written informed consent. The research protocol strictly followed the principles of the Helsinki Declaration and was approved by the Humanities and Ethics Committee of the Affiliated Hospital of Jining Medical University (2018-FY-070).

### Direct DNA Sequencing of the ATG16L1 Gene Promoter

Peripheral leukocytes were isolated, and genomic DNA was extracted with the DNeasy Blood and Tissue Kit (QIAGEN, Valencia, CA, United States). From the Genebank database, 1,246 bases upstream of the transcription start point of the *ATG16L1* gene (NCBI: NC_000002.12) were selected for analyses, and PCR primers for the *ATG16L1* gene promoter were designed from the sequence. Two overlapping DNA fragments were generated: fragment 1, 233,250,476 to 233,251,194 (719 bp), and fragment 2, 233,251,120 to + 233,251,721 (602 bp), and these were synthesized by Shanghai Sangon Biotech. All PCR primers are shown in Table 1. The ATG16L1 gene promoter was amplified by polymerase chain reaction using two pairs of primers. The amplified target fragments were then sent to Shanghai Sangon Biotech for Sanger Sequencing. High-throughput sequencing was performed using Illumina HiSeq2500/4000 sequencing platform.

**TABLE 1:** PCR primers for the human ATG16L1 gene promoter.

**Primers**	**Sequences**	**Location**	**Products (bp)**
Sequencing			
ATG16L1-F1	5′-TTCATCTCCCCCTTTCAACA-3′	233250476	719 bp
ATG16L1-R1	5′-GCTTGGTACAGGGGAAACCT-3′	233251194	
ATG16L1-F2	5′-ATGCTCCTGCTGTCAGGGTA-3′	233251120	602 bp
ATG16L1-R2	5′-GAGCTCACCTCCACACACTG-3′	233251721	
Functioning			
ATG16L1-F	5′-(*Kpn*I)-CCCAAACAAACCACAAAACC-3′	233250648	1,075 bp
ATG16L1-R	5′-(*Hin*dIII)-GGAGCTCACCTCCACACACT-3′	233251722	

*PCR primers were designed based on the genomic DNA sequence of the ATG16L1 gene (NC_000002.12). The transcription start site (TSS) is at position 233251571 (+ 1).*

### Functional Analyses With the Dual Luciferase Assay

By means of the sequence comparison between the sample and wild type for preliminary screening, we designed primers to amplify the *ATG16L1* gene promoter region (1,075 bp, from -923 bp to + 152 bp) that contains the variant site fragment, and we added *Kpn*I and *Hin*dIII loci at both ends (Table 1). The expression constructs were generated by subcloning PCR products into the *Kpn*I and *Hin*dIII sites of a reporter vector-pGL3-basic, expressing the luciferase gene. The plasmid containing Renilla luciferase gene (PhRM-TK) was used as the control plasmid and reporter plasmid to provide internal control of transcription efficiency, so that the test results could not be interfered by the changes of experimental conditions. We selected three cell lines, human embryonic cardiac fibroblasts (HEH2), rat cardiomyocytes (H9c2), and human embryonic kidney cells (HEK-293), to detect the transcription activity of the *ATG16L1* gene promoter. The expression vector (pGL3 basic) without the *ATG16L1* gene promoter sequence was used as the negative control for transfection efficiency. Expression constructs expressing the Renilla luciferase gene (pRL-TK) were used as internal controls in the HEK-293 (30 ng), HEH2 (60 ng), and H9c2 (60 ng) cells. The day before liposome transfection, HEH2, HEK-293, and H9c2 cells were evenly seeded into 6-well plates. The next day, we transfected 0.5 μg of the designated expression construct into a 6-well plate where the HEK-293 cells were grown, and 1.0 μg designated expression structure was transfected into the HEH2 and H9c2 cell growth 6-well plates, respectively. The medium was replaced 5 h after transfection. The transfected cells were harvested after a specific amount of time (36 h for HEK-293 cells and 48 h for the HEH2 and H9c2 cells). The double luciferase activity of the transfected cells was detected by the dual luciferase reporter system (Promega Dual-Luciferase^®^ Reporter Assay system). The transcription activity of the *ATG16L1* gene promoter was represented as a ratio of luciferase to Renilla luciferase activity. The transcription activity of the wild-type *ATG16L1* gene promoter was set to 100%. The whole experiment including transfection were repeated independently at least three times.

### Preparation of Nuclear Extracts and the Electrophoretic Mobility Shift Assay

Nuclear extracts of the HEK-293 and H9c2 cells were prepared with an NE-PER^®^ Nuclear Protein/Cytoplasmic Protein Extraction Kit (Thermo Fisher Scientific, Inc.). The protein concentration of the nuclear extract was determined using Bio-Rad Protein Assay Reagent and stored at –80°C until use. Biotinylated double-stranded oligonucleotides (30 bp) were used that contained SNP sites as probes. A LightShift^®^ chemiluminescence electrophoretic mobility shift assay (EMSA) kit (Thermo Fisher Scientific, Inc.) was used for the DNA-protein binding reaction to explore the interaction between the DNA fragment of *ATG16L1* gene promoter and the nucleoprotein. Unfortunately, due to the limitations of EMSA experimental technology, it is impossible to determine whether the protein directly interacts with the polymorphic sites on the promoter or indirectly affects the transcription process of the gene.

### Statistical Analyses

SPSS25.0 software was used for statistical analyses of clinical data. Quantitative data are expressed as means ± SEMs and were analyzed using a Student’s *t*-test, and qualitative data were tested using the chi-square test. The Hardy--Weinberg equilibrium test was used to analyze the allele distribution of the SNPs. A chi-square test was performed to evaluate significant differences in the allele and genotype frequencies. Logistic regression was used to analyze the odds ratios (ORs) and 95% confidence intervals (CIs) in the measurement of the allele correlations between SNPs and AMI. Using the web-based software SNP-Stats^1^, five genetic models (dominant, dominant, dominant, recessive, and log-additive) were analyzed after adjusting for age and sex. We used the HaploView software package (version 4.2) to analyze the SNP linkage disequilibrium (LD) between the two groups, and we used the SHE-sis software platform^2^ to perform haplotype analyses of LDs and associations based on haplotype. To explore SNP--SNP interactions and select the model that had the maximum cross-validation consistency, the General Multi-Factor Dimensionality Reduction (GMDR) software package (version 0.9) was used. The web browser program Genetic Association Study Power Calculator^3^ was used to compute statistical power. The TRANSFAC database was used to predict the relevant transcription factors for *ATG16L1* gene promoter polymorphism. Values of *P* < 0.05 were considered to indicate statistical significance.

## Results

### Clinical Characteristics

The demographic and clinical characteristics of the AMI patients and controls are shown in Table 2. The mean age of the controls was 45.76 ± 12.77 years, and the group included 237 males (63.30%) and 92 females (36.70%). The mean age of the patients was 63.80 ± 12.27 years old, and the group included 229 males (75.94%) and 130 females (24.06%). Compared with controls, AMI patients had a significantly higher prevalence of the traditional risk factors such as male, older age, smoking history, hypertension, and diabetes (*P* = 0.021, *P* = 0.000, *P* = 0.000, *P* = 0.000, *P* = 0.000, respectively). The BMI of AMI group was significantly lower than that of control group (*P* = 0.011), possibly due to the influence of various environmental factors such as age and gender. Levels of total cholesterol (TC) and low-density lipoprotein (LDL) were significantly higher in the AMI group than in the control group, while HDL showed the opposite relationship. Statistical analyses showed that there were significant differences in TC (*P* = 0.000), HDL-C (*P* = 0.000), and LDL-C (*P* = 0.00) between the two groups. There were no significant differences in systolic blood pressure, diastolic blood pressure, or triglycerides between the two groups (*P* = 0.155, *P* = 0.791, *P* = 0.561), which may have been the result of drug administration.

**TABLE 2:** Characteristics of the study population.

**Parameters**	**AMI cases (*n* = 329)**	**Controls (*n* = 359)**	***P*-value**
Male/female (n)	237/92	229/130	0.021
Age (years)	63.80 ± 12.27	45.76 ± 12.77	0.000
Smoking [n (%)]	172 (52.4)	59 (16.4)	0.000
Hypertension [n (%)]	149 (45.3)	87 (24.2)	0.000
Diabetes [n (%)]	74 (22.5)	28 (7.8)	0.000
BMI (kg/m2)	24.78 ± 3.76	26.49 ± 3.61	0.011
SBP (mmHg)	125.98 ± 23.04	128.18 ± 17.42	0.155
DBP (mmHg)	78.64 ± 15.36	78.36 ± 11.96	0.791
HDL-C (mmol/L)	1.07 ± 0.42	1.32 ± 0.30	0.000
LDL-C (mmol/L)	2.49 ± 0.80	2.81 ± 0.73	0.000
TG (mmol/L)	1.49 ± 0.96	1.44 ± 1.08	0.561
TC (mmol/L)	4,28 ± 1.08	4.95 ± 1.43	0.000

*SBP, systolic blood pressure; DBP, diastolic blood pressure; HDL-C, high-density lipoprotein cholesterol; LDL-C, low-density lipoprotein cholesterol; TG, triacylglycerol; TC, total cholesterol, (P < 0.05) was statistically significant.*

### DNA-Sequence Variants in Acute Myocardial Infarction Patients and Controls

**FIGURE 1:**
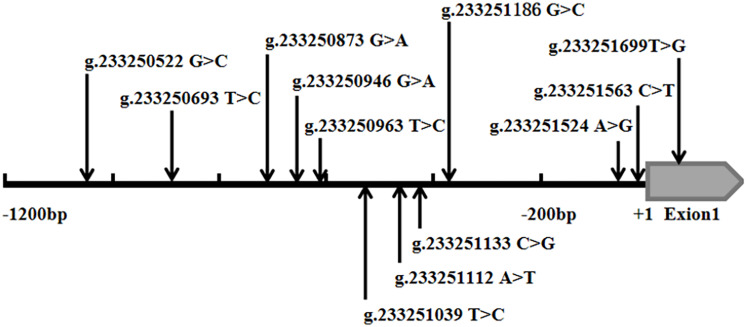
Schematic diagram of gene mutation site of ATG16L1 gene promote. The DSVs and SNPs in the ATG16L1 gene promoter identified in AMI patients and controls. Schematic representation of the DSVs within the ATG16L1 gene promoter. The numbers represent the genomic DNA sequences of the human ATG16L1 gene (Genbank accession number NC_000002.12) upstream to the transcription start site (+1).

In the sequence detection of PCR fragments in the promoter region of the *ATG16L1* gene in 690 subjects, a total of 12 DSVs, including 10 SNPs, were identified (Table 3). Their locations are shown in Figure 1, and the oligonucleotide sequences of three of them are presented in Table 4. Statistical analyses were performed on all DSVs. Three SNPs [g.233250693 T > C (rs185213911), g.233250946 G > A (rs568956599), and g.233251133 C > G (rs1301744254)] were identified in five AMI patients and were not found in controls. We also identified AMI patients with three SNPs [g.233250693 T > C (rs185213911), g.233250946 G > A (rs568956599), and g.233251699 T > G (rs2289477)]. In addition, in two AMI patients with the SNP g.233251133 C > G (rs1301744254), one was not accompanied by the SNP g.233251699 T > G (rs2289477), and the other was. The SNP g.233251699 T > G (rs2289477) was also found in other AMI patients and controls.

**TABLE 3:** DSVs within the ATG16L1 gene promoters in AMI patients and controls.

**Polymorphisms**	**Genotypes**	**Location**	**Controls (*n* = 359)**	**AMI (*n* = 329)**	***P*-value**
g.233250522 G > C (rs75824126)	GC	−1049 bp	12	21	0.062
g.233250693 T > C (rs185213911)	TC	−878 bp	0	2	−
g.233250873 G > A (rs146693112)	GA	−698 bp	6	6	0.879
g.233250946 G > A (rs568956599)	GA	−625 bp	0	1	−
g.233250963 T > C (rs1816753)	TT	−608 bp	88	64	0.187
	TC		171	177	
	CC		100	88	
g.233251039 T > C (rs12476635)	TT	−532 bp	308	277	0.345
	TC		47	51	
	CC		4	1	
g.233251112 A > T (rs74599577)	AT	−460 bp	12	21	0.062
g.233251133 C > G (rs1301744254)	CG	−438 bp	0	2	−
g.233251186 G > C	GC	−385 bp	1	0	−
g.233251524 A > G	AG	−47 bp	1	0	−
g.233251563 C > T (rs77820970)	CT	−8 bp	1	0	−
g.233251699 T > G (rs2289477)	TT	129 bp	131	129	0.631
	TG		179	152	
	GG		49	48	

*DSVs were located upstream to the transcription start site of ATG16L1 gene at 233251571 of NC_000002.12.*

**TABLE 4:** The double-stranded biotinylated oligonucleotides for the EMSA.

**DSVs**	**Oligonucleotide**	**Locations**
g.233250693T > C (rs185213911)	5′-TTCAGTGGGACACTC(T/C) TCCCAACGCACCCT-3′	233250678–233250707
g.233250946G > A (rs568956599)	5′-GGAAAGTCTCTGGCC(G/A) GAGGGGAGGCTATC-3′	233250931–233250961
g.233251133C > G (rs1301744254)	5′-CAATGCTCCTGCTGT(C/G) AGGGTAGGCCTTGG-3′	233251118–233251147

**FIGURE 2:**
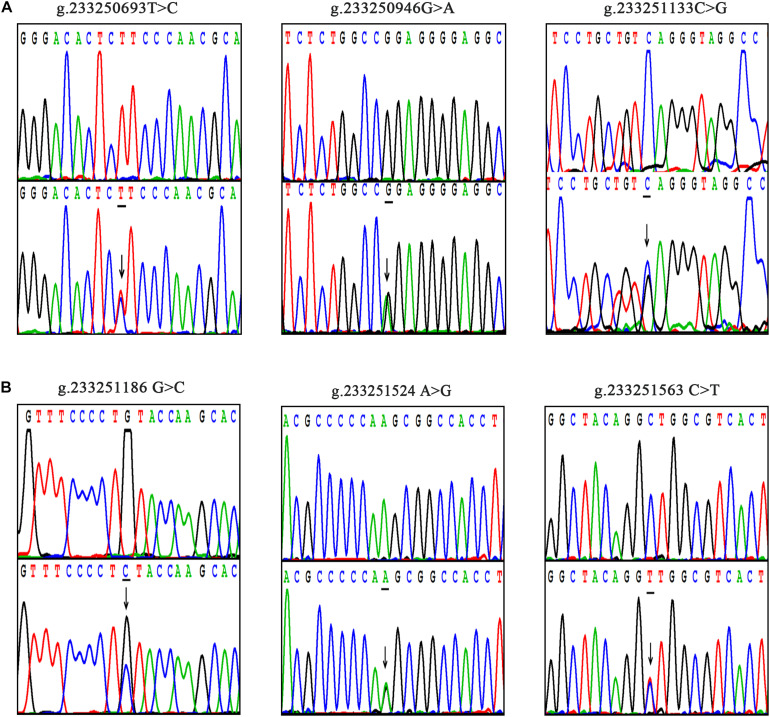
Sequencing chromatograms of the DSVs and SNPs within the ATG16L1 gene promoter in AMI patients and controls. All sequence orientations of the DSVs and SNPs are forward. **(A)** Sequencing chromatograms of the SNPs in AMI patients. **(B)** Sequencing chromatograms of the DSVs and SNP in healthy controls. The top layers are wild-type sequences, the bottom panels are heterozygous mutations, and the mutation sites are marked with arrows.

The DNA-sequencing chromatograms of two novel DSVs and four SNPs are shown in Figure 2. The SNPs identified in AMI patients are shown in Figure 2A, and the DSVs and SNPs identified in controls are presented in Figure 2B. In addition, six SNPs [g.233250522 G > C (rs75824126), g.233250873 G > A (rs146693112), g.233250963 T > C (rs1816753), g.233251039 T > C (rs12476635), g.233251112 A > T (rs74599577), and g.233251699 T > G (rs2289477)] were identified in both AMI patients and controls at similar frequencies (*P* > 0.05). The sequencing chromatograms of these are not shown.

### Correlation Analyses of Single-Nucleotide Polymorphisms in the ATG16L1 Gene Promoter and Acute Myocardial Infarction

In this study, 10 SNPs were found in the two groups. The allelic distribution and minor allele frequencies (MAFs) of the SNPs in the two groups are given in Table 5. Nine SNPs (rs75824126, rs185213911, rs146693112, rs568956599, rs1816753, rs12476635, rs74599577, rs1301744254, and rs2289477) were found in 329 AMI patients. Statistical analyses were performed for all SNPs. The Hardy–Weinberg equilibrium test indicated that all SNPs in the two groups showed Hardy–Weinberg equilibrium (*P* > 0.05), indicating that the samples were from a population with genetic balance and had good representativeness. The MAFs of the 10 SNPs ranged from approximately 0 to 50% in both AMI patients and controls. No associations were observed, however, between the 10 SNPs and AMI. We screened nine SNP loci in the *ATG16L1* gene promoter for analyses, and their genotypic distributions were consistent with Hardy–Weinberg equilibrium in both the case group and the control group. There were no statistically significant differences in the frequency of genotypic distribution among cases and controls. The web-based software SNP-Stats was used to evaluate the correlations among multiple genetic models of SNPs and AMI, after correcting for age and sex in unconditional logistic regression analyses. As shown in Table 6, the genotype distribution of the SNP rs75824126 conforms to the recessive genetic model (OR = 0.57; 95% CI = 0.36–0.92, *P* = 0.02, AIC = 642.3) and the overdominant model (OR = 1.49; 95% CI = 1.01–2.19, *P* = 0.043, AIC = 643.7), although the recessive model is better (has a smaller AIC value). The SNP rs12476635 genotype distribution conforms to the codominant model (OR = 1.49; 95% CI = 1.01–2.19, *P* = 0.02–3.84, AIC = 643.7) and the overdominant model (OR = 1.87; 95% CI = 1.07–3.27, *p* = 0.027, AIC = 642.9), and the overdominant model is better (has a smaller AIC value). However, the differences in genetic models for SNP (rs2289477) had no statistical significance (*P* > 0.05).

**TABLE 5:** Genotype, the minimum allele frequency of variation, genotype distribution and Hardy–Weinberg equilibrium test in SNPs and crude odds ratio estimates for AMI risk.

**SNPs**	**Position**	**Alleles(A/B)**	**Controls (*n* = 359)**			**HWE**	**AMI (*n* = 329)**			**HWE**	**MAF**		**OR (95%CI)**	***P*-value**
			**AA**	**AB**	**BB**	***P*-value**	**AA**	**AB**	**BB**	***P*-value**	**Controls**	**AMI**		
rs75824126	233,250,522	G/C	347	12	0	0.747	308	21	0	0.550	0.017	0.032	0.507 [0.245∼1.048]	0.067
rs185213911	233,250,693	T/C	359	0	0	1.000	327	2	0	0.959	0.000	0.003	–	–
rs146693112	233,250,873	G/A	353	6	0	0.873	323	6	0	0.867	0.008	0.009	0.915 [0.292∼2.866]	0.879
rs568956599	233,250,946	G/A	359	0	0	1.000	328	1	0	0.978	0.000	0.002	–	–
rs1816753	233,250,963	T/C	88	171	100	0.380	64	177	88	0.138	0.517	0.536	0.929 [0.750∼1.149]	0.496
rs12476635	233,251,039	T/C	308	47	4	0.158	277	51	1	0.398	0.081	0.081	0.948[0.642∼1.400]	0.787
rs74599577	233,251,112	A/T	347	12	0	0.747	308	21	0	0.550	0.017	0.032	0.507 [0.245∼1.048]	0.067
rs1301744254	233,251,133	C/G	359	0	0	1.000	327	2	0	0.956	0.000	0.003	–	–
rs2289477	233,251,699	T/G	131	179	49	0.324	129	152	48	0.767	0.386	0.377	1.039 [0.834∼1.295]	0.732

**TABLE 6:** Genetic models’ analysis of the association between the SNPs in the ATG16L1 promoter region and AMI (adjusted by SEX + AGE).

**SNP ID**	**Model**	**Genotype**	**Control**	**AMI**	**OR (95%CI)**	***P*-value**	**AIC**	**BIC**
rs1816753	Codominant	C/C	99 (27.6%)	88 (26.8%)	1.00	0.043	643.5	666.1
		T/C	171 (47.8%)	176 (53.7%)	1.24 (0.79–1.95)			
		T/T	88 (24.6%)	64 (19.5%)	0.66 (0.38–1.14)			
	Dominant	C/C	99 (27.6%)	88 (26.8%)	1.00	0.93	647.8	665.9
		T/C-T/T	259 (72.3%)	240 (73.2%)	1.02 (0.67–1.56)			
	Recessive	C/C-T/C	270 (75.4%)	264 (80.5%)	1.00	0.02	642.3	660.4
		T/T	88 (24.6%)	64 (19.5%)	0.57 (0.36–0.92)			
	Overdominant	C/C-T/T	187 (52.2%)	152 (46.3%)	1.00	0.043	643.7	661.8
		T/C	171 (47.8%)	176 (53.7%)	1.49 (1.01–2.19)			
	Log-additive	−	−	−	0.83 (0.63–1.09)	0.19	646	664.2
rs12476635	Codominant	T/T	307 (85.8%)	276 (84.2%)	1.00	0.26 (0.02–3.84)	643.8	666.4
		T/C	47 (13.1%)	51 (15.6%)	1.85 (1.06–3.24)			
		C/C	4 (1.1%)	1 (0.3%)	0.26 (0.02–3.84)			
	Dominant	T/T	307 (85.8%)	276 (84.2%)	1.00	0.058	644.2	662.3
		T/C-C/C	51 (14.2%)	52 (15.8%)	1.69 (0.98–2.91)			
	Recessive	T/T-T/C	354 (98.9%)	327 (99.7%)	1.00	0.26	646.5	664.6
		C/C			0.24 (0.02–3.50)			
	Overdominant	T/T-C/C	311 (86.9%)	277 (84.5%)	1.00	0.027	642.9	661
		T/C	47 (13.1%)	51 (15.6%)	1.87 (1.07–3.27)			
	Log-additive	−	−	−	1.47 (0.89–2.42)	0.13	645.5	663.6
rs2289477	Codominant	T/T	131 (36.6%)	128 (39%)	1.00	0.91	649.6	672.2
		T/G	179 (50%)	152 (46.3%)	0.92 (0.61–1.40)			
		G/G	48 (13.4%)	48 (14.6%)	0.91 (0.50–1.67)			
	Dominant	T/T	131 (36.6%)	128 (39%)	1.00	0.67	647.6	665.7
		T/G-G/G	227 (63.4%)	200 (61%)	0.92 (0.62–1.37)			
	Recessive	T/T-T/G	310 (86.6%)	280 (85.4%)	1.00	0.88	647.7	665.9
		G/G	48 (13.4%)	48 (14.6%)	0.96 (0.55–1.67)			
	Overdominant	T/T-G/G	179 (50%)	176 (53.7%)	1.00	0.76	647.7	665.8
		T/G	179 (50%)	152 (46.3%)	0.94 (0.64–1.38)			
	Log-additive	−	−	−	0.95 (0.71–1.26)	0.7	647.6	665.7

*OR, odds ratio; 95% CI, confidence intervals; AIC, Akaike’s information criterion; BIC, Bayes’s information criterion.*

### Power Calculation

We used the Genetic Association Study Power Calculator (see text footnote 3) to compute statistical power. The power calculation was conducted with various parameters. For example, the ratio of cases (*n* = 330) to controls (*n* = 360) was 0.917, and the significance level of the study design was set to *P* < 0.05. After analyzing recent epidemiological reports on cardiovascular disease in China, we set disease prevalence to 0.10, and the multiplication model was chosen for disease risk. The disease allele frequency was approximately 0.0076 (5/660). We assumed that the relative genotype risks were 1.5, 3.0, and 3.2, respectively. Cases + controls were the independent variables used to plot against statistical power. Finally, the values for statistical power were 0.127, 0.727, and 0.803, respectively.

### Linkage Disequilibrium and Haplotype Analyses

**FIGURE 3:**
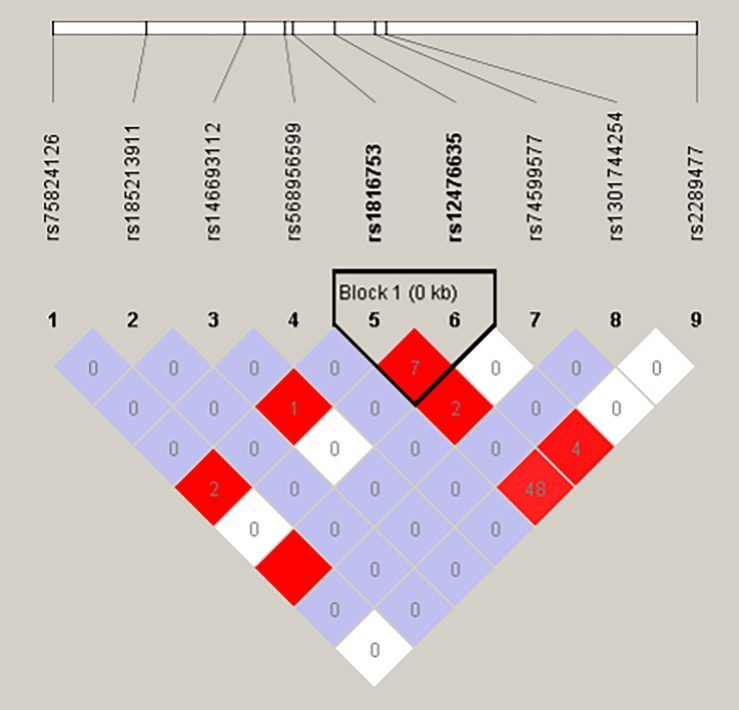
The LD tests of the Nine SNPs in AMI. Standard color schemes indicate different levels of LD. Standard Color Scheme: White: LOD < 2, D′ < 1; Blue: LOD < 2, D′ = 1; Bright red, LOD > 2, D′ = 1; LOD: the log of the likelihood odds ratio, is a measure of confidence in the value of D′. Shown LD value: R-squared. The box is empty indicates that the r2 value is 1.000.

We used the HaploView software package (version 4.2) and the SHE-sis software platform to analyze the LDs and haplotypes of six SNPs in the *ATG16L1* gene promoter region. The two SNPs (rs75824126 and rs74599577) in the promoter region of the *ATG16L1* gene showed a perfect LD (D′ = 1.000, r2 = 1.000) and the SNPs (rs1816753 and rs2289477) were in an LD (D′ > 0.800, r2 > 0.330) (Figure 3). The SNPs rs1816753 and rs12476635 made up LD Block 1. As shown in Table 7, in the haplotype analysis region, no one haplotype of the *ATG16L1* promoter had a significant difference in frequency distribution between the AMI group and the control group.

**TABLE 7:** Haplotype analysis of the SNPs in the ATG16L1 promoter region and the association with AMI risk.

**SNP1**	**SNP2**	**SNP3**	**SNP4**	**SNP5**	**SNP6**	**SNP7**	**SNP8**	**SNP9**	**AMI (freq)**	**Control (freq)**	**Chi2**	**Fisher’s P**	**OR (95%CI)**
C	T	G	G	C	C	T	C	T	0.00 (0.000)	1.07 (0.001)	−	−	−
C	T	G	G	C	T	T	C	T	19.92 (0.030)	10.93 (0.015)	3.536	0.060070	2.018 [0.957∼4.253]
G	T	A	G	C	C	A	C	T	0.10 (0.000)	1.64 (0.002)	−	−	−
G	T	A	G	T	T	A	C	G	0.00 (0.000)	2.06 (0.003)	−	−	−
G	T	A	G	T	T	A	C	T	5.90 (0.009)	2.30 (0.003)	−	−	−
G	T	G	G	C	C	A	C	G	0.00 (0.000)	1.06 (0.001)	−	−	−
G	T	G	G	C	C	A	C	T	52.90 (0.080)	51.23 (0.074)	0.392	0.531055	1.136 [0.7762∼1.695]
G	T	G	G	C	T	A	C	G	239.92 (0.365)	264.23 (0.368)	0.024	0.877675	0.983 [0.788∼1.226]
G	T	G	G	C	T	A	C	T	35.25 (0.054)	40.85 (0.057)	0.076	0.782678	0.937 [0.589∼1.490]
G	T	G	G	T	T	A	C	G	3.18 (0.005)	9.65 (0.013)	−	−	−
G	T	G	G	T	T	A	C	T	294.74 (0.448)	332.99 (0.464)	0.388	0.533203	0.934 [0.754∼1.158]
C	T	G	G	C	T	T	C	G	1.08 (0.002)	0.00 (0.000)	−	−	−
G	C	G	G	C	T	A	C	G	2.00 (0.003)	0.00 (0.000)	−	−	−
G	T	G	A	C	T	A	C	G	0.82 (0.001)	0.00 (0.000)	−	−	−
G	T	G	A	T	T	A	C	T	0.18 (0.000)	0.00 (0.000)	−	−	−
G	T	G	G	T	T	A	G	G	1.00 (0.002)	0.00 (0.000)	−	−	−
G	T	G	G	T	T	A	G	T	1.00 (0.002)	0.00 (0.000)	−	−	−

*SNP (1-9): rs75824126, rs185213911, rs146693112, rs568956599, rs1816753, rs12476635, rs74599577, rs1301744254, rs2289477.*

### Prediction of Transcription Factors Within the Polymorphism Sequence

The TRANSFAC database is the most comprehensive database for collecting information on transcription factors and transcription factor binding sites. We selected it to obtain information on *ATG16L1* transcription factors in *Homo sapiens*. The key to the regulation of gene expression lies in the regulation of the transcription initiation level, so it is important to study the effects of *ATG16L1* gene promoter mutation on transcription factors. We found that the SNPs in the promoter region of *ATG16L1* may change the binding of the transcription factors. As indicated in Table 8, the SNPs [233250693 T > C (rs185213911), 233250946 G > A (rs568956599), 233251133 C > G (rs1301744254)] found in AMI patients can create, modify, and eliminate putative binding sites for transcription factors, which may affect the binding of transcription factors to gene promoters, thereby affecting transcription levels and autophagy function, and may lead to the occurrence and development of disease. Some healthy individuals may develop such disease when exposed to environmental factors and already carrying those significant variants.

**TABLE 8:** Predicted binding sites for transcription factors affected by the SNPs.

**SNPs**	**Change mode**	**Transcription factors**
g.233250693T > C (rs185213911)	Create	NF-kappaB, GKLF
	Weaken	ETS1
	Abolish	RBP-Jkappa,TCF-1
g.233250946G > A (rs568956599)	Create	PPARgamma, PPARalpha, VDR
	Enhance	T3R-beta
	Weaken	AP-2alpha,CP2
	Abolish	Elk-1, Fli-1,ETS1
g.233251133C > G (rs1301744254)	Create	ZIC3,Zbtb44,SREBP–1/2
	Weaken	c-MAF
	Abolish	meis1,HDAC1,ATF-4,CTCF

*NF-kappaB, Nuclear Factor-kappaB; GKLF, gut-enriched Kruppel-like factor; Ets1, E26 transformation specific-1; RBP-jkappa, Recombination Signal Binding Protein-jkappa; TCF-1, T cell factor-1; PPARalpha, peroxisome proliferator-activated receptor alpha; PPARgamma, Peroxisome proliferator-activated receptor gamma; VDR, Vitamin D receptor; T3R, thyroid hormone receptor; AP-2alpha, activator protein 2alpha; SREBP, sterol regulatory element-binding protein; CREB1, cAMP responsive element binding protein 1; c-MAF, Serum-derived Macrophage Activating Factor; HDAC1, histone deacetylase 1 gene; ATF-4, Activated transcription factor 4; CTCF, CCCTC binding factor.*

The SNP g.233250693 T > C (rs185213911) may create the binding sites for NF-kappaB, GKLF, weaken the binding sites for ETS1, and abolish the binding site for RBP-Jkappa,TCF-1. Nuclear factor-kappaB (NF-kappaB) is an important regulator of immunity and inflammation, and it is a typical pro-inflammatory signal transduction factor (Lawrence, 2009; Bartuzi et al., 2013). The inflammatory activation of endothelial cells by the NF-κB activator Ikk2 promotes the development and progression of atherosclerosis (Mussbacher et al., 2020). Ets-1 is a potentially important regulator of DNA repair mechanisms. The proliferation of vascular smooth muscle cells (VSMCs) plays an important role in the pathogenesis of atherosclerosis and restenosis. Transcription factor GKLF can induce inhibition of VSMC proliferation (Wassmann et al., 2007, 2010). Studies have found that when both Ets1 and Ets2 are mutated, endothelial cell apoptosis is significantly increased both *in vivo* and *in vitro*. Mutations in ts1 and Ets2 can lead to embryonic death in the second trimester, and obvious vascular-branch defects are observed (Wei et al., 2009; Chetty and Sumanas, 2020). Studies have shown that in mice with endothelial-specific RBP-jkappa mutations, the activity of lipase and the transendothelial transport of long-chain fatty acids to muscle cells are impaired (Jabs et al., 2018). TCF-1 plays an important role in mammalian T cells (Raghu et al., 2019).

The SNP g.233250946 G > A (rs568956599) may create binding sites for peroxisome proliferator-activated receptor (PPAR) gamma (PPARgamma), PPAR alpha (PPARalpha), and VDR; enhance the binding sites for T3R-beta; weaken the binding sites for AP-2alpha and CP2; and abolish the binding site for Elk-1, Fli-1, and ETS1. PPAR plays a role in the regulation of lipid and glucose metabolism. PPARgamma has an important role in the regulation of gene expression in various diseases including obesity, diabetes, and cancer (Janani and Ranjitha Kumari, 2015). A large amount of research indicates the importance of PPARalpha for the transcriptional regulation of lipid metabolism, atherosclerosis, and inflammation (Duval et al., 2007; Zandbergen and Plutzky, 2007; Bougarne et al., 2018; Yamashita et al., 2019). VDR plays an important role in the regulation of energy metabolism. Overexpression of VDR in adipose tissue leads to an increase in body weight, fat mass, and serum levels of lipids, as well as a decrease in energy metabolism (Xu et al., 2019a). AP-2-alpha deficiency can cause malformations of the outflow tract of the embryonic heart (Brewer et al., 2002). The expression of ELK-1 protein in the atrial tissue of patients with chronic AF is significantly reduced, indicating that the downregulation of the expression of transcription activator ELK-1 may play an important role in the pathogenesis of AF (Zeng et al., 2015). In a previous study (Hart et al., 2000), mice with null mutations in the Fli-1 locus died on day 11.5 of embryogenesis due to loss of vascular integrity and loss of the Fli-1 hemizygotes that cause megakaryocyte defects. Under physiological and diabetic conditions, Ets1 inhibits gluconeogenesis, and hepatocyte Ets1 knockout mice have enhanced hepatic glucose production (Li et al., 2019); Ets1 also plays an important role in regulating the differentiation and survival of endothelial cells and promoting the development of blood vessels (Chetty and Sumanas, 2020).

The SNP g.233251133 C > G (rs1301744254) may create binding sites for ZIC3, Zbtb44, and SREBP-1/2; weaken the binding sites for serum-derived macrophage activating factor; and abolish the binding sites for meis1, HDAC1, activated transcription factor 4 (ATF-4), and CCCTC binding factor (CTCF). ZIC3 can bind to and inhibit cardiac α-actin promoter through its zinc finger domain (Zhu et al., 2007). ZBTB44 may regulate the formation of new blood vessels through the cZBTB44-miR-578-VEGFA/VCAM1 axis and participate in related diseases (Zhou J. et al., 2020). Cholesterol synthesis and catabolism pathways are regulated by transcription driven by SREBPs (Sato, 2020). Sulfonic acid reduces the precursor and mature form of SREBP-1/2 in the liver of HFD-fed rats, and it can prevent the hepatic steatosis caused by HFD (Morsy et al., 2020). c-MAF enhances the phagocytic activity of macrophages by enhancing their phagocytic efficiency (Kawakatsu et al., 2019). Meis1 can cooperate with Hoxb13 to regulate the maturation and cell cycle of cardiomyocytes (Nguyen et al., 2020). Under hypoxia, knocking down MEIS1 induces PASMC proliferation and migration (Locatelli et al., 2020; Yao et al., 2020). HDAC1 reduces pathological vascular calcification both *in vivo* and *in vitro* (Zhou R. M. et al., 2020). ATF-4 is induced by translation under hypoxic conditions and mediates part of the unfolded protein response after ER stress (Köditz et al., 2007). CTCF is a highly conserved, ubiquitous zinc finger protein, and it is related to many important cellular processes, such as transcription activation, repression, insulation, imprinting, and promotion of DNA double-strand break repair (Tanwar et al., 2019).

### Functional Analyses of DNA-Sequence Variants by Dual-Luciferase Reporter Assay

The effects of DSVs on the transcriptional activity of the *ATG16L1* promoter were detected by constructing a luciferase reporter gene vector containing the following wild-type and variant *ATG16L1* promoters: wild-type pGL3-WT, pGL3-233250693C, pGL3-233250873A, pGL3-233250946A, pGL3-233251039C, pGL3-233251111T, pGL3-233251133G, pGL3-233251186G, pGL3-233251524C, pGL3-233251563T, and pGL3-233251699G. These expression vectors were transiently transfected into HEH2, HEK-293, and H9c2 cells to detect dual luciferase activity. The transcriptional activity of *ATG16L1* gene promoter mutants was compared with that of wild type (100%).

**FIGURE 4:**
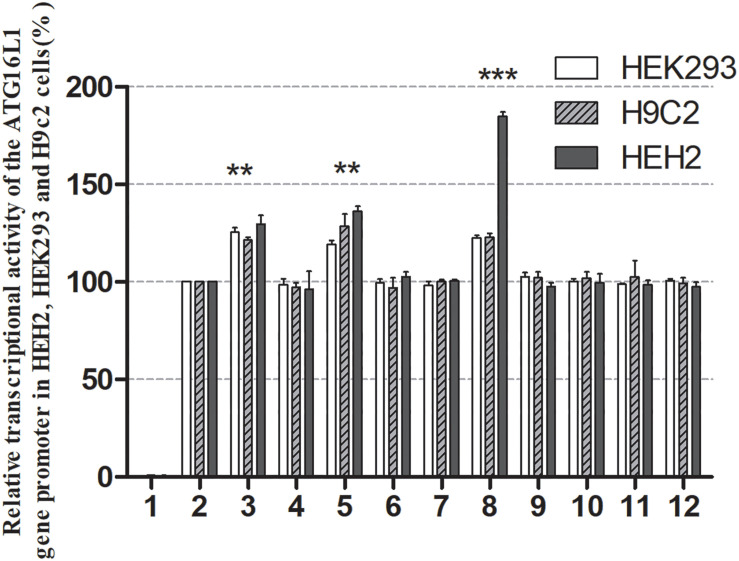
Relative transcriptional activity of wild-type and variant ATG16L1 gene promoters. Wild-type and variant ATG16L1 gene promoters were cloned into reporter gene vector pGL3 and transfected into cultured cells. The transfected cells were collected, and dual-luciferase activities were assayed. Empty vector pGL3-basic was used as a negative control. The transcriptional activity of the wild-type ATG16L gene promoter was designed as 100%. The relative activities of the ATG16L1 gene promoter were calculated. Relative activities of wild-type and variant ATG16L1 gene promoters in HEK-293, H9c2 and HEH2 cells. Lanes 1, pGL3-basic; 2, pGL3-WT; 3, pGL3-233250693C; 4, pGL3-233250873A; 5, pGL3-233250946A; 6, pGL3-233251039C; 7, pGL3-233251111T; 8, pGL3-233251133G; 9, pGL3-233251186G; 10, pGL3-233251524C; 11, pGL3-233251563T, and 12, pGL3-233251699G. **p < 0.01; ***p < 0.001.

Three SNPs [233250693 T > C (rs185213911), 233250946 G > A (rs568956599), 233251133 C > G (rs1301744254)] were identified only in the AMI group and significantly increased the transcriptional activity of the *ATG16L1* gene promoter relative to the wild-type *ATG16L1* gene promoter in HEH2, HEK-293, and H9c2 cells (*P* < 0.05). The others did not (*P* > 0.05) (Figure 4). Two novel heterozygous DSVs (g.233251186 G > C, g.23321524 A > G) and one SNP [g.233251563 C > T (rs77820970)] were only detected in the control and did not affect the transcriptional activity of the *ATG16L1* gene promoter, unlike the wild-type *ATG16L1* gene promoter (*P* > 0.05). Five SNPs [g.233250873 G > A (rs146693112), g.233250963 T > C (rs1816753), g.2332511039 T > C (rs12476635), 233251111A > T (rs74599577), and 233251699 T > G (rs2289477)], which were found in both AMI patients and controls, did not affect the transcriptional activity of the *ATG16L1* gene promoter compared with the wild-type *ATG16L1* gene promoter (*P* > 0.05). Three SNPs [g.233250693 T > C (rs185213911), g.233250946 G > A (rs568956599), g.233251133 C > G (rs1301744254)] identified in AMI patients significantly altered the expression level of luciferase in HEH2, HEK-293, and H9c2 cells, the difference in the degree of influence may be due to tissue specific expression.

### Binding Sites for Transcription Factors Affected by DNA-Sequence Variants

**FIGURE 5:**
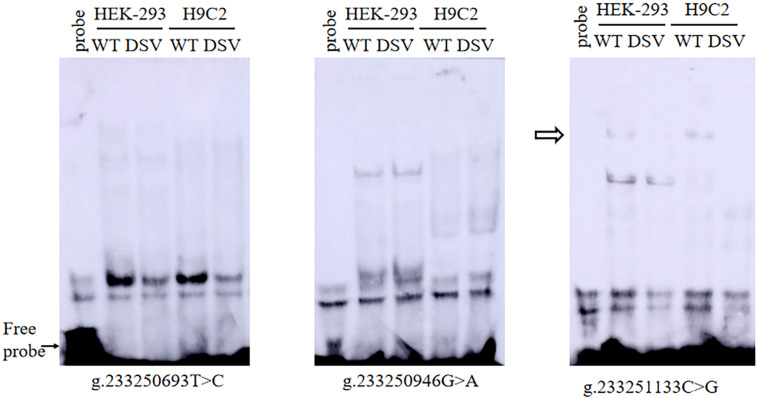
EMSA of SNPs biotin-labeled oligonucleotides in AMI patients. Wild-type and mutant oligonucleotides (30 bp) were designed, and SNPs of AMI patients were labeled with biotin, including [g.233250693 T>C (rs185213911), g.233250946 G>A (rs568956599), and g.233251133 C>G (rs1301744254)]. EMSA was conducted with biotinylated oligonucleotides and the nuclear extracts from HEK-293 and H9c2 cells. The free probe was marked with an arrow at the bottom. The affected binding for transcription factors was marked with an open arrow.

To further assess the effects of DSVs and SNPs on the binding sites of the transcription factor binding site of the *ATG16L1* gene promoter, EMSA was compared with wild-type and variant oligonucleotides. The nucleotide sequences of the DSVs and SNPs are shown in Table 4. The EMSA results showed that the SNP g.233250693 T > C (rs185213911) weakened binding to transcription factors, while the SNP g.233251133 C > G (rs1301744254) eliminated the binding of a transcription factor to the *ATG16L1* gene promoter in both HEK-293 and H9c2 cells (Figure 5). Therefore, it is speculated that these two SNPs identified in AMI patients may affect the transcription level of the *ATG16L1* gene by interfering with the binding of the transcription factor with the *ATG16L1* gene promoter.

## Discussion

There have hitherto been no genetic analyses of *ATG16L1* gene promoter in CAD or AMI. In this study, we used a large-sample, case-control study to conduct genetic and functional analyses of the *ATG16L1* gene promoter in AMI patients and healthy controls. In humans, most genetic variation in this area is rare, and rare and low-frequency variation in coding might have a large effect on human phenotypes (Lettre, 2014). *ATG16L1* is an essential protein in the early stages of autophagy. In cardiovascular disease, studies have shown that *ATG16L1* expression can affect vascular endothelial function in atherosclerosis (Magné et al., 2015). In this study, a total of two novel DSVs and 10 SNPs were found in AMI patients and controls. We identified three SNPs [233250693 T > C (rs185213911), 233250946 G > A (rs568956599), 233251133 C > G (rs1301744254)] in AMI patients and one SNP [233251133 C > G (rs1301744254)] that significantly altered the transcriptional activity of the *ATG16L1* gene. The SNP [g.233250693 T > C (rs185213911)] may create the binding sites for NF-kappaB, GKLF, weaken the binding sites for ETS1, and abolish the binding site for RBP-Jkappa,TCF-1. The SNP [g.233250946 G > A (rs568956599)] may create the binding sites for PPARgamma, PPARalpha, VDR; enhance the binding sites for T3R-beta; weaken the binding sites for AP-2alpha, CP2, and abolish the binding site for Elk-1, Fli-1, ETS1. The SNP [g.233251133 C > G (rs1301744254)] may create the binding sites for ZIC3, Zbtb44, SREBP-1/2, weaken the binding sites for c-MAF, and abolish the binding site for meis1, HDAC1, ATF-4, CTCF. These mutations may affect the binding of transcription factors to gene promoters, thereby affecting transcription levels and autophagy function, and may lead to the occurrence and development of disease. Further EMSA revealed that this SNP affects the binding of transcription factors. The collective frequency of the SNPs in the *ATG16L1* gene promoter was 2.11% (7/329) in AMI patients. The three SNPs as low-frequency risk factors, combined with environmental factors, may promote the occurrence and development of AMI.

A database search revealed that *ATG16L1* maps to the forward strand of chromosome 2q37.1, 233251571–233295674, which contains 19 exons, spans more than 43.9 kb of genomic DNA, and is ubiquitously expressed in human tissues. The protein encoded by this gene is part of a large protein complex that is necessary for autophagy, the major process through which intracellular components are targeted to lysosomes for degradation. ATG16 and ATG12–ATG5 form a protein complex of about 800 kDa, which is released from the membrane before or after the completion of autophagosomes, so it is a good marker of isolation membranes. Membrane recruitment of the autophagy complex is a key step in autophagy. The plasma membrane of the ATG5-binding fragment ectopically recruited to *ATG16L1* leads to abnormal LC3 and structural lipid metabolism (Fujita et al., 2008). The absence of *ATG16L1* disrupts the recruitment of ATG12–ATG5 conjugates for separation membranes, resulting in the LC3 of microtubule-associated protein 1 coupled with phosphatidylethanolamine. In *ATG16L1*-deficient cells, the formation of autophagy and the degradation of long-lived proteins are seriously damaged (Saitoh et al., 2008). One study suggested that the *ATG16L1* complex is a novel E3-like enzyme that can dynamically locate the putative source membrane formed by autophagy and act as a scaffold for LC3 lipids (Rai et al., 2019). The Atg12–5-16L1 complex is a necessary condition for phagocytic cell elongation, and the absence of Atg5 or *ATG16L1* in mice has been found to completely eliminate the formation of autophagosomes (Kuma et al., 2004). Human Apgl6L is a coiled-coil protein and has a large C-terminal domain containing seven WD repeats (Zheng et al., 2004). This CCD can be used to form homo-oligomer. WD repeats form a three-propeller structure, which provides a stable platform for simultaneous interactions with multiple proteins (Smith et al., 1999). *ATG16L1* requires highly conserved sequences within its CCD to homodimerize. The conserved residues in CCD mediate direct binding to phosphoinositide, including 3-phosphatidylinositol (PI3P), thereby enhancing its PAS localization and autophagy. This indicates that *ATG16L1* has the inherent ability to bind autophagy-related membranes through direct interaction with PI3P (Dudley et al., 2019). However, although the importance of *ATG16L1* in autophagy has been recognized, its precise function and mechanisms remain to be studied and elucidated.

CAD and AMI are chronic inflammatory and metabolic diseases that are mainly caused by atherosclerosis. The AMI is associated with metabolism disorder, lipid oxidation, and overweight, specifically with those with a smoking history. In this study, it was found that the prevalence of AMI patients with male, advanced age, smoking history, hypertension, diabetes, and other traditional risk factors was significantly higher than that of the control group. The levels of total cholesterol and low density lipoprotein in AMI group were significantly higher than those in control group, while the levels of high density lipoprotein were opposite. These are consistent with the traditional perception of AMI risk factors. The BMI of AMI patients was lower than that of the control group, possibly due to the influence of age. Therefore, a healthy lifestyle and effective control of weight, blood pressure, blood lipids and blood glucose can help to reduce or delay the occurrence of AMI. Atherosclerotic plaque can initiate autophagy under lipid oxidation, inflammation, and metabolic stress, and can protect plaque cells from oxidative damage by degrading damaged intracellular substances (Martinet and De Meyer, 2009; Swiader et al., 2016; Osonoi et al., 2018). Autophagy may be an important physiological or pathophysiological response to cardiac stress, such as ischemia or pressure overload in patients with CAD, hypertension, aortic valve disease, and congestive heart failure (Levine and Kroemer, 2008). Autophagy dysfunction is associated with abnormal lipid metabolism and inflammation, and autophagy deficiency may play a role in some inherited heart diseases (e.g., Danone’s disease and Pompe’s disease). Autophagosome accumulation has been found in cardiac biopsies and isolated stress cardiomyocytes of patients with these diseases (Terman and Brunk, 2005; Linton et al., 2015; Yan et al., 2015; Yamaguchi, 2019). Some studies have shown that autophagy dysfunction can lead to muscle disease. The failure of autophagosomes and lysosomes to fuse leads to vacuolar myopathy in rats and humans (Bolaños-Meade et al., 2005; Kassan et al., 2016). Pompe’s disease, caused by a deficiency of glycogenic glycemic enzyme acid α-glucosidase, results from the accumulation of autophagosomes (Fukuda et al., 2006). Previous studies have demonstrated that the expression of *ATG16L1* in atherosclerosis is closely related to the instability of lesions on atherosclerotic plaques (Wang and Ye, 2014). In the necrotic core and surrounding area of human atherosclerotic plaque, particularly the shoulder area, *ATG16L1* protein is abundantly expressed, and *ATG16L1* immunomarkers are mainly found in the plaque endothelial cells and foamy smooth muscle cells. The expression of *ATG16L1* enhances the autophagy of macrophages and has been shown to prevent atherosclerosis (Leng et al., 2016; Orsatti et al., 2018). The association of the human *ATG16L1* gene with the occurrence and development of AMI remains to be confirmed in future studies.

In summary, we found that SNPs identified in AMI patients significantly alter the transcriptional activity of the ATG16L gene promoter. Therefore, we speculate that these SNPs may change the level of *ATG16L1*, promoting the occurrence and development of AMI through autophagic and non-autophagic functions as low-frequency risk factors.

## Conclusion

This was the first study to find that the *ATG16L1* gene promoter polymorphism is associated with AMI. Genetic variation in the *ATG16L1* gene promoter may be an important factor determining the genetic susceptibility of AMI individuals. To confirm that this polymorphism is a new genetic marker for the occurrence and development of AMI and plaque rupture, it is necessary to investigate it in larger populations and in other ethnic groups.

## Data Availability Statement

The original contributions presented in the study are included in the article/supplementary material, further inquiries can be directed to the corresponding author/s.

## Ethics Statement

The studies involving human participants were reviewed and approved by the Humanities and Ethics Committee of the Affiliated Hospital of Jining Medical University. The patients/participants provided their written informed consent to participate in this study.

## Author Contributions

FH and BY conceived, designed the experiments, and wrote the manuscript. FH and SP performed the experiments. FH, ZS, and YC analyzed the data. All authors read and approved the final manuscript.

## Conflict of Interest

The authors declare that the research was conducted in the absence of any commercial or financial relationships that could be construed as a potential conflict of interest.

## Footnotes

1 https://www.snpstats

2 http://analysis.bio-x.cn/SHEsisMain.htm

3 http://csg.sph.umich.edu/abecasis/gas_power_calculator/index.html
